# Flexible terahertz beam manipulation and convolution operations in light-controllable digital coding metasurfaces

**DOI:** 10.1016/j.isci.2024.111688

**Published:** 2025-01-03

**Authors:** Min Jia, Chao Zhao, Hui Wang, Weiran Sun, Yuncheng Lu

**Affiliations:** 1 Communication Research Center, School of Electronics and Information Engineering, Harbin Institute of Technology, Harbin 150080, China

**Keywords:** Natural sciences, Physics, Applied sciences

## Abstract

The development of coding metasurface opens an important development direction for beam modulator components. However, the lack of dynamic tunability is a major obstacle to the development and practicality of terahertz coding metasurface. In this work, we design and implement a wireless optically controlled tunable coding metasurface by integrating photosensitive silicon into the metasurface, which realizes the ultra-fast dynamic switching effect of terahertz beam and dynamic convolution operation of 2-bit coding metasurface. We also achieve dynamic modulation of vortex electromagnetic waves based on wireless light-controllable coding metasurfaces. And the genetic algorithm is used to reverse design the array arrangement of the coding metasurface to achieve terahertz broadband radar scattering cross-section reduction. The proposed optically controlled dynamic metasurface developed in this study has the potential to create non-contact metasurfaces, thereby making it a valuable tool for future practical applications.

## Highlights

• Wireless light-controllable metasurface is proposed for dynamic control of terahertz beam
• The dynamic convolution operation of 2-bit coding metasurface is realized
• Dynamic modulation of the orbital angular momentum (OAM) beam is achieved
• Terahertz broadband radar cross-section (RCS) reduction is achieved

## Graphical abstract

**Figure:**
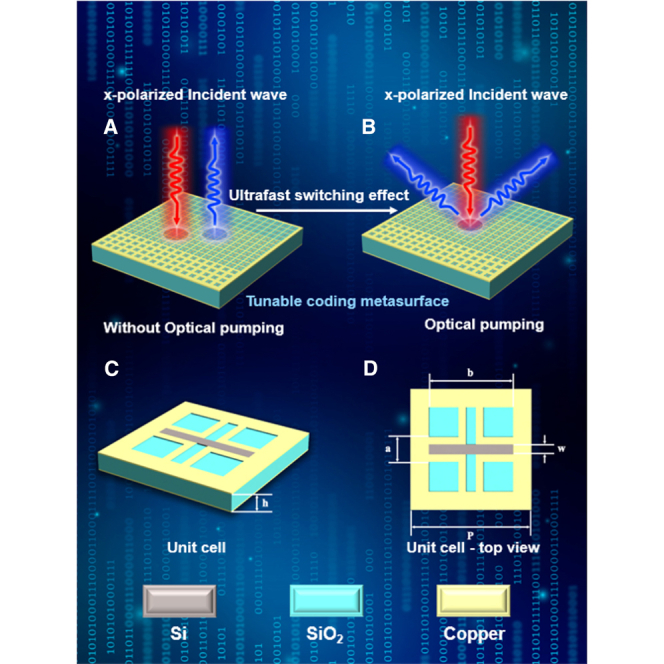

## Introduction

The terahertz band, with a frequency between microwave and infrared, has a very large bandwidth due to its high frequency and can provide extremely high information transmission rates.^1^^,^^2^^,^^3^^,^^4^^,^^5^ Terahertz-related technologies are crucial for advancing next-generation high-speed communications,^6^^,^^7^ high-resolution imaging,^8^^,^^9^ intelligent sensory integration,^10^^,^^11^ and other information fields. However, the terahertz beam is narrow and difficult to aim accurately. Traditional mechanical beam manipulation is slow and lacks integration, while phased array systems are complex and expensive. Thus, there is an urgent need to develop high-performance beam manipulation devices. Metasurfaces are a novel type of structures and devices that are crucial for achieving this function.^5^^,^^12^ A metasurface is an ultrathin composite structure with specific electromagnetic control functions.^13^^,^^14^ It is formed by arranging subwavelength artificial units on a two-dimensional surface by certain array guidelines. In 2011, Professor Capasso from Harvard University proposed a generalized Snell’s law, breaking the intrinsic design method of metasurfaces.^15^ Researchers have been inspired by the concept of phase mutation to design metasurfaces. This approach has produced a series of high-quality research results, contributing greatly to the revolutionary development of metasurfaces.^16^^,^^17^^,^^18^^,^^19^ Metasurfaces have already demonstrated considerable potential in many applications, including electromagnetic stealth,^20^^,^^21^^,^^22^ polarization conversion,^23^^,^^24^ antennas,^25^^,^^26^^,^^27^ and imaging.^28^ However, once a conventional analog metasurface has been fabricated, its fixed topological geometry restricts it to performing only one or a few specific electromagnetic functions. It is not possible to dynamically change the functions according to actual requirements.^29^ Thus, there is an immediate requirement to investigate new characterization options for the conventional metasurface research system, as well as metasurface design with dynamically tunable, real-time programmable design.

In 2014, Cui et al. innovatively introduced the concept of digital coding into metamaterial design, proposing a “digital version” of metamaterials and metasurfaces.^30^ The idea of digital coding allows researchers to design and study metasurfaces from a completely new perspective in information science, constructing a new research system.^31^ Dynamic modulation of plane waves in the microwave band was achieved using programmable metasurfaces. Tunable metasurfaces gained attention from researchers and were quickly developed.^32^^,^^33^^,^^34^^,^^35^ The introduction of dynamic modulation mechanisms to the metasurfaces will promote the diversification of its electromagnetic functions and further extend its range of applications and scenarios. In recent years, numerous attempts have been made to realize metasurfaces with controlled amplitude and phase modulation using tunable materials and novel designs.^36^ The method used by the researchers to achieve efficient vector field manipulation in the terahertz band using a transmission-based three-layer metasurface is expected to advance fields, such as terahertz communication and optical information security.^37^ Some researchers have realized the identification and reconstruction of spectral and polarization states, which provides a new method for optical information collection and processing.^38^ The researchers propose an all-optically controlled non-logic gate based on a metasurface double-layer, which achieves directional asymmetric transmission. This design realizes efficient manipulation of optical signals through the interaction of two layers on the metasurface, and provides new possibilities for optical computing and optical information processing.^39^

Currently, the most important tunable materials include liquid crystals,^40^^,^^41^^,^^42^ two-dimensional materials,^43^^,^^44^^,^^45^ and phase change materials.^46^^,^^47^ The main modulation mechanisms of the metasurface include mechanical modulation, temperature modulation, electrical modulation, optical modulation, and modulation of micro/nanoelectromechanical systems. In the microwave range, electrically controlled diodes and varactor diodes can be used but hardly work in the terahertz frequency range. Electrically tuned dynamic metasurfaces are typically connected to external power and control circuits through physical wires. The electrical control method can cause crosstalk between signals, which can negatively impact the performance of the metasurface.^48^^,^^49^ Additionally, the size of the entire system may increase, particularly when a large number of wires are required for complex control. Thermally tuned dynamic metasurfaces typically exhibit slow response speeds and limited modulation ranges, rendering them unsuitable for applications with stringent temperature requirements. This limitation has become a major bottleneck in their development.^50^^,^^51^ The studies aforementioned indicate that tunable materials, such as vanadium dioxide, liquid crystals, and graphene, require sensitive temperature or feedthrough conditions, making them challenging to apply in practical settings. Few attempts have been made in the aforementioned studies to investigate the convolution theorem for achieving dynamic scattering pattern shifts in active terahertz coding metasurfaces.^52^^,^^53^^,^^54^^,^^55^ Therefore, it is crucial to investigate the manipulation of tunable and reconfigurable terahertz beams using the convolution theorem in active dynamic metasurfaces that can be easily and efficiently tuned.

In this work, we propose a tunable coding metasurface to realize real-time dynamic tuning of terahertz beams. The integration of the photosensitive silicon on the surface of the coding element is realized by a hybrid layer of metal pattern and photosensitive silicon, a dielectric layer, and a metal grounding layer. Photosensitive silicon is a material whose conductivity alters with the energy of the pump light. It is simple to regulate and possesses a fast response time. The wireless optically tunable coding metasurface has been designed to enable ultra-fast dynamic switching effects for the dynamic convolutional operation of 2-bit coding metasurfaces. Our design for optically tuned dynamic metasurfaces is expected to excel in future practical applications due to its non-contact tuning capabilities.

### Design of tunable coding metasurfaces

**Figure 1:**
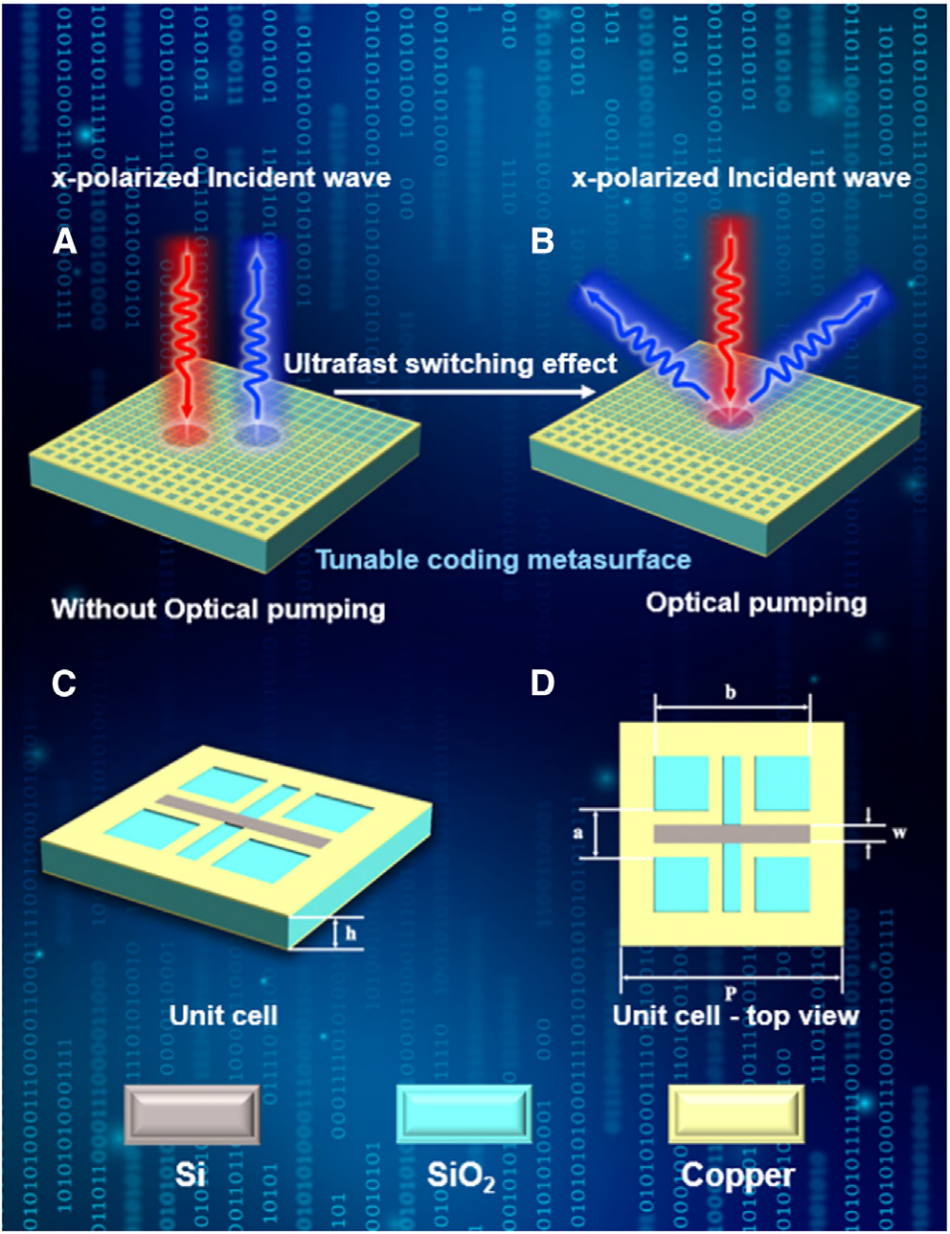
Schematic of the tunable coding metasurface (A and B) Conceptual illustration of beam switching of normal and anomalous reflection at a coding metasurface. (C) 3D diagram of the unit. (D) Top view of the unit.

As illustrated in Figures 1A and 1B, in order to reduce the size of the tunable terahertz coding metasurface, while simultaneously simplifying the tunable mechanism and improving the fabrication process, we propose a single-layer microstructured photosensitive silicon coding metasurface, which can be fully modulated by light intensity. As shown in Figures 1C and 1D, the structure of the metasurface unit comprises three layers of material with a unit period of p=40μm. The top layer is made up of photosensitive silicon and metallic copper, with a thickness of 0.2μm. The middle dielectric layer is silicon dioxide, with a dielectric constant of 3.9 and a thickness of h=10μm. The bottom layer is metallic copper, with a thickness of 0.5μm. For the metal part of the top layer, where the parameter a is 8μm. The photosensitive silicon is embedded in the metal structure with a dielectric constant of 11.5, a width of w=3μm, and a variable length b. The parameters a and w are the same for subsequent 1-bit and 2-bit unit structures. The phase of the reflected electromagnetic wave can be modulated by varying the structural parameters b.

As a common engineering material, copper is also a good conductive metal. Copper materials have high plasticity, good forging, and tensile properties. These exemplary process characteristics render copper an accessible material for the manufacture of metamaterials, which can satisfy the demands of intricate processes and diminish the complexity and expense of production. Copper is a material with a wide range of applications in the field of metamaterials, with reflective metasurface units representing a particularly common use case. Silicon dioxide exhibits a high refractive index in specific frequency bands, including the terahertz band. This property makes it one of the preferred dielectric materials for the design and realization of metamaterials. The elevated refractive index facilitates the capacity of metamaterials to influence electromagnetic waves, including phase modulation and beam forming. The use of silicon dioxide in metamaterial design allows for greater flexibility and tunability, thereby meeting the demand for dielectric properties in a variety of application scenarios. The conductivity of photosensitive silicon is susceptible to significant fluctuations in response to changes in light conditions. The optoelectronic tunability of photosensitive silicon enables its dynamic tuning in metamaterial design, thus meeting the varying electromagnetic property demands of different application scenarios. The preparation process of photosensitive silicon is relatively mature, and high-quality photosensitive silicon materials can be prepared by a variety of methods. This facilitates a reduction in the cost of preparing metamaterials and an improvement in production efficiency.

**Figure 2:**
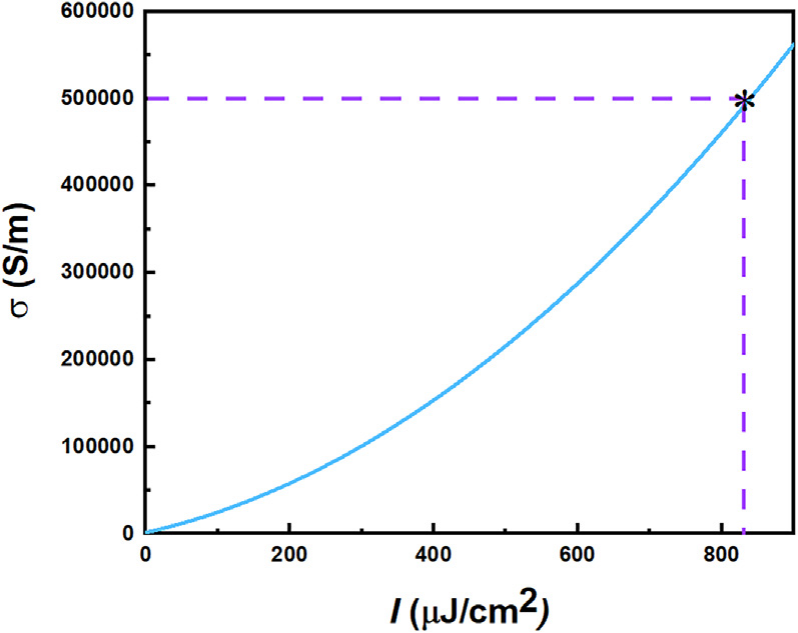
The relationship between the intensity of pump light and the conductivity of the photosensitive silicon

The conductivity of photosensitive silicon can be modulated by the energy of the pump light. When the light energy increases, the carrier concentration within the semiconductor also increases, causing a change in conductivity. The conductivity σ of photosensitive silicon is related to the pump power I by the equation $\sigma =4.863\times 10^{-4}\times I^{2}+0.1856\times I+1.569$. Photosensitive silicon is a material with a sensitive response in the THz band and is commonly used in the design and fabrication of THz wave modulation devices. Photosensitive silicon is a material that exhibits a sensitive response in the THz band, and it is frequently employed in the design and fabrication of THz wave modulation devices. Figure 2 illustrates the impact of pump light intensity on the conductivity of photosensitive silicon. At no pumping excitation, the conductivity of the photosensitive silicon is 0. When the pump power is $790\;\mu J/{\text{cm}}^{2}$, the conductivity increases to $5.0\times 10^{5}S/m$.

The far-field scattering patterns function expression for a vertically incident plane wave on a coding metasurface is:

(Equation 1) $$f\left(\theta ,\phi \right)=f_{e}\left(\theta ,\phi \right)\sum_{m=1}^{M}\sum_{n=1}^{N}\exp\left\{-i\left\{\phi \left(m,n\right)+\frac{2\pi }{\lambda }d\;\sin\;\theta \left[\left(m-\frac{1}{2}\right)\cos\;\phi +\left(n-\frac{1}{2}\right)\sin\;\phi \right]\right\}\right\}$$

Where θ and ϕ are the polar and azimuth angles, $f_{e}\left(\theta ,\phi \right)$ is the scattering pattern of, ϕ(m,n) is the scattering phase, where the d represents the period of a single unit in the metasurface. The formula for the deflection angle θ of the coded sequence is as follows:

(Equation 2) θ=arcsin(λ/Γ)

Where λ represents the wavelength of the operating frequency and Γ represents the period of the coding sequence. By designing the period length of the coding sequence, the Angle of abnormal refraction of the THz beam can be regulated, so as to achieve the purpose of modulating the THz beam.

## Results and discussions

### 1-bit light-controllable digital coding metasurface

To design the 1-bit tunable coding metasurface, we only need two units. When designing the photosensitive silicon length b to be either b=32.5μm or b=38μm, a phase difference of around 180° is achieved. As a result, we utilize the tunable unit with b=32.5μm as the “0” element and the tunable unit with b=38μm as the “1” element.

**Figure 3:**
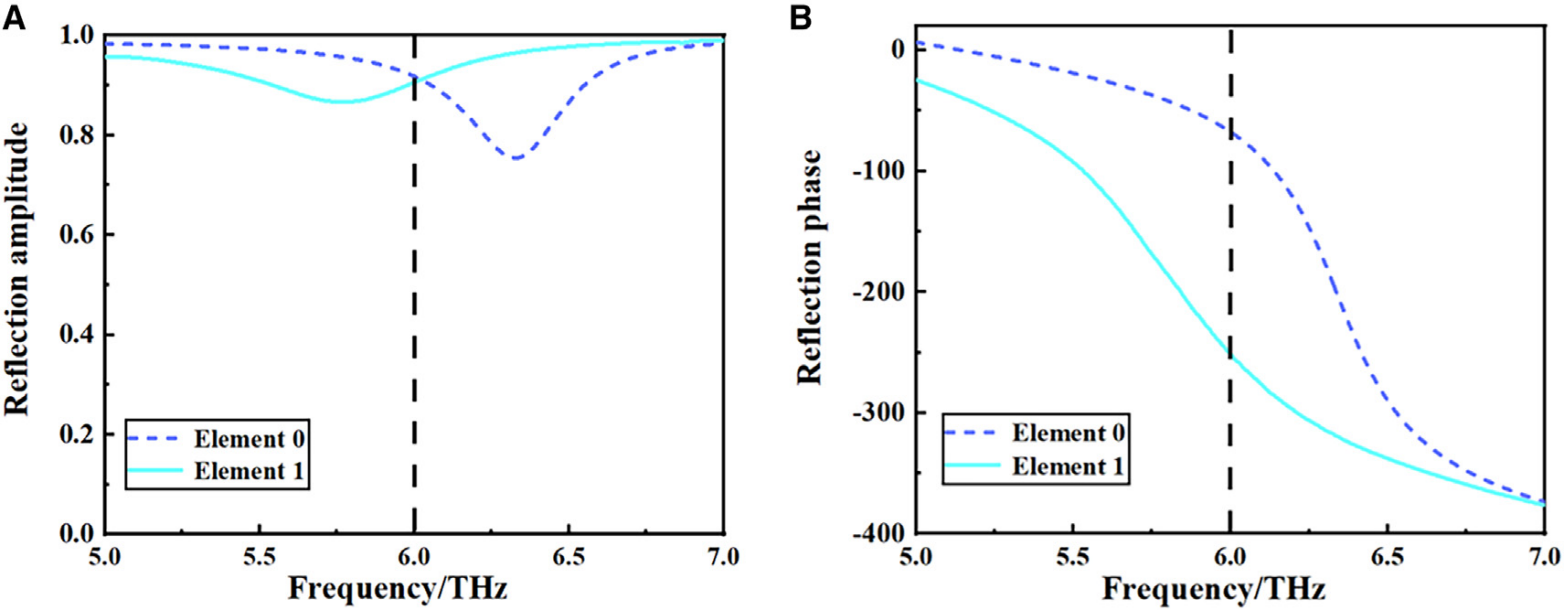
The two digital elements of 1-bit tunable coding metasurfaces and their reflection response curves (A) The reflection amplitude of the 1-bit digital units from 5 to 7 THz. (B) The reflection phase of the 1-bit digital units from 5 to 7 THz.

The corresponding phase curves of the two digital units over the frequency band from 5 to 7 THz are presented in Figure 3. The resulting phase responses of the two elements are analyzed in Table 1. As illustrated in Figure 3A, the amplitudes of the “0” and “1” structural units exhibit high reflection at 6 THz, offering a robust foundation for far-field simulations. The phases of the unitary structures are illustrated in Figure 3B, and the phases of the “0” and “1” units are −67.5° and ˗247.7°, respectively, with a phase difference of approximately 180° between the two structures. It can be observed that the correlation properties of the reflectivity and phase of the two units designed are in accordance with the requirements of the corresponding coding metasurface units.

**Table 1:** 1-bit digital elements of tunable coding metasurfaces

Phase response over frequency at 6 THz (1-bit)		
Code	“0”	“1”
Phase[deg]	−67.5	−247.7

**Figure 4:**
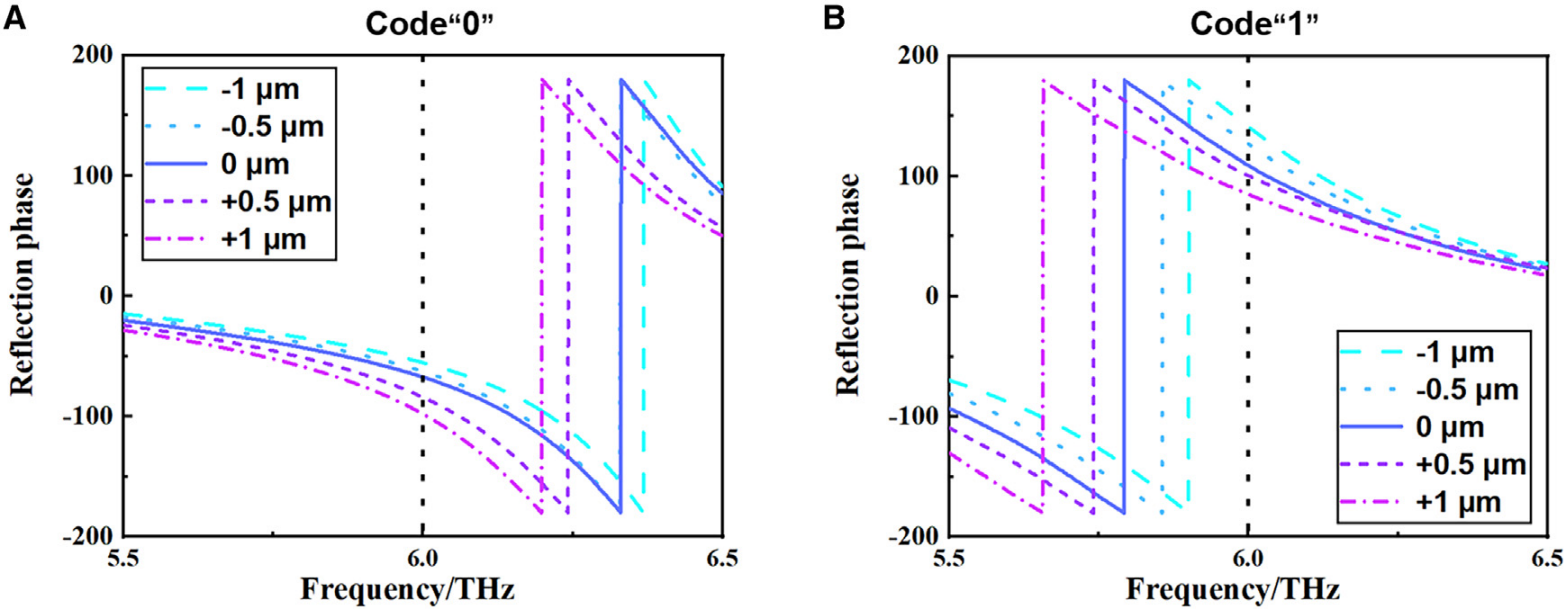
Tolerance analysis of geometrical parameters of photosensitive silicon (A) The reflection phase of the 1-bit digital units code“0” from 5.5 to 6.5 THz. (B) The reflection phase of the 1-bit digital units code“1” from 5.5 to 6.5 THz.

As the devices are subject to some variation in process manufacturing, it is necessary to investigate the effect of process variation on their performance. We have carried out a tolerance analysis, which helps the experiment in many ways. Tolerance analysis allows the experimental designer to determine the allowable range of variation for each experimental variable, i.e., the tolerance range. This allows more robust experimental protocols to be designed by taking into account the effect of variation in these variables on the experimental results within the experimental design. Given that the fundamental construct of the unit configuration put forth in this paper is the active material photosensitive silicon, an error analysis is conducted on the variable parameter b pertaining to the photosensitive silicon. As illustrated in Figure 4, the reflection phase curves of 1-bit cells “0” and “1” are presented. The parameters b of the units “0” and “1” are 32.5μm and 38μm, respectively, on the basis of which they are assigned an error of ±0.5μm and ±1μm, respectively. In the context of 1-bit coding metasurfaces, the phase difference continues to demonstrate satisfactory functionality and performance in the majority of practical applications when situated within the range of 160°–200°. As can be observed in Table 2, the phase generation error is within the permissible range and has essentially no effect on the performance of the coding metasurfaces. This indicates that the device designed in the paper is somewhat tolerant.

**Table 2:** Phase difference with ±3% variation of photosensitive silicon geometric parameters

Variation	−1μm	−0.5μm	0μm	+0.5μm	+1μm
Code “0” Phase[deg]	−55.3	−62.5	−71	−84	−97
Code “1” Phase[deg]	−218.9	−235.8	−249.6	−262.7	−275
Phase Difference[deg]	162.7	173.3	178.6	178.7	178

Different modulation functions of electromagnetic waves can be achieved by emitting these coding units in a specific coding sequence on a two-dimensional plane. To minimize the coupling effect, this paper adopts the super-unit cell design method, where the size is set as a super-unit cell composed of N×N identical units. Here, the size of the super unit cell is set to 4×4 and the coding patterns are all composed of 32×32 coding cells. The 1-bit tunable coding metasurface example was modeled and simulated using the commercial software CST Microwave Studio. The software employs plane wave excitation modes and open boundary conditions.

**Figure 5:**
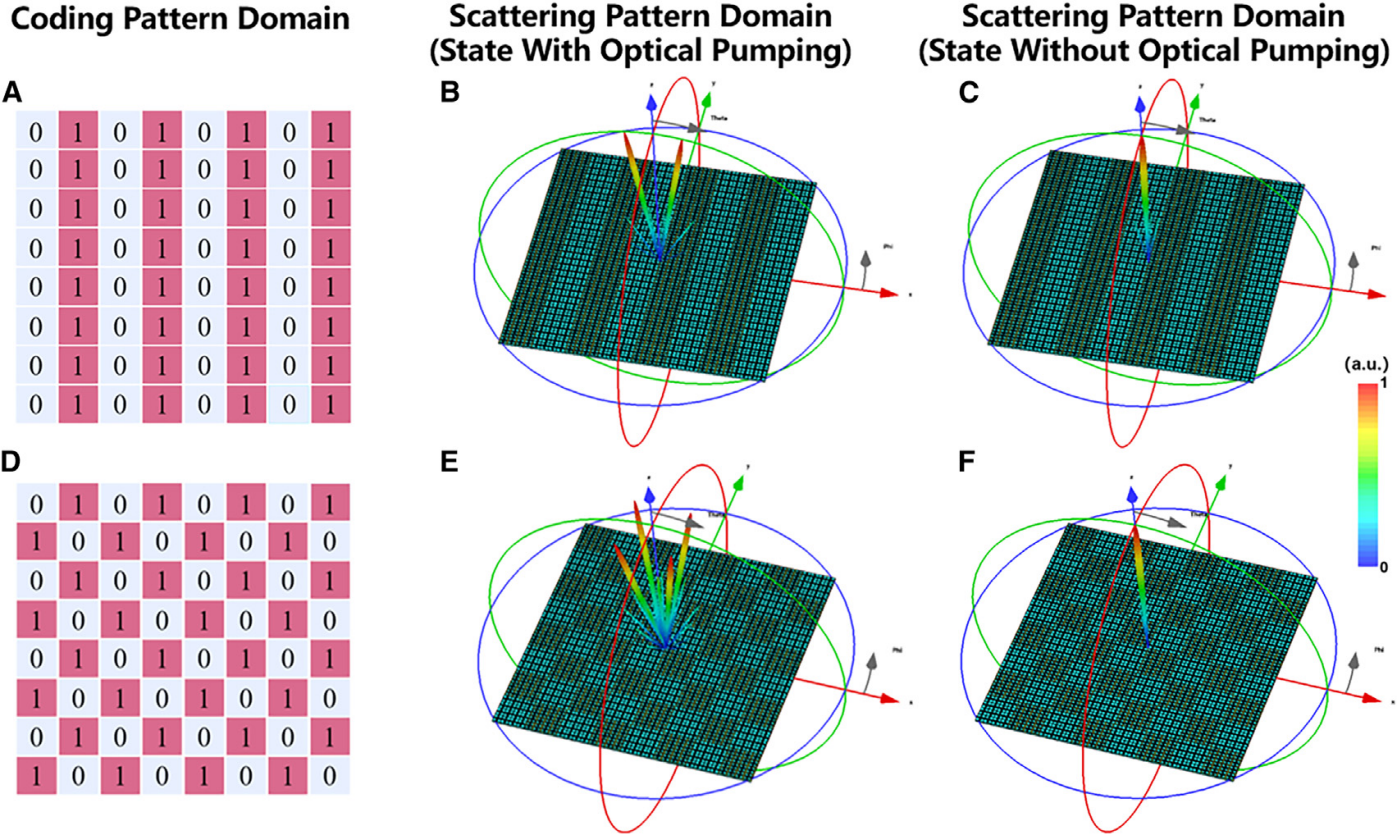
Schematic of and the switching effect of simulated 3D scattering patterns at a 1-bit light-controllable coding metasurface (A) Schematic of the coding sequence 0101 …/0101 … at 6 THz. (B) 3D scattering pattern with optical pumping of the coding sequence in Figure 5A. (C) 3D scattering pattern without optical pumping of the coding sequence in Figure 5A. (D) Schematic of the coding sequence 0101 …/1010 … at 6 THz. (E) 3D scattering pattern with optical pumping of the coding sequence in Figure 5D. (F) 3D scattering pattern without optical pumping of the coding sequence in Figure 5D.

Figure 5A displays the schematic diagram of the coding sequence “0101/0101”, which comprises units “0” and “1”. Figures 5B and 5C illustrate the far-field 3D scattering patterns before and after the function switch. When the metasurface is in an optically pumped state, its conductivity is $5.0\times 10^{5}S/m$. At 6 THz, the perpendicular incident beam is mainly reflected by the metasurface in two directions symmetrically along the y axis, and the result of the 3D scattering pattern is shown in Figure 5B. The reflected beams peak at −9° and 9°, indicating a deflection angle of 9°. By substituting Γ=320μm and λ=50μm into the formula, we can calculate the deflection angle to be 8.9°. The 3D far-field diagrams confirm that the simulation is consistent with the theoretical calculation. When the metasurface is in the absence of optical pumping, its conductivity is 0S/m. Figure 5C demonstrates that a plane wave, with a frequency of 6 THz, generates a vertical beam in the opposite direction to the incident wave when it strikes the metasurface. Similarly, Figure 5D shows a tessellated coding metasurface composed of cells “0” and “1” that form the coding sequence “0101/1010”. Figures 5E and 5F display the far-field diagrams before and after the function switching. When the metasurface is in the state of optical pumping, the main beam pointing to the z axis transforms into four anomalous reflective beams symmetrically emitted. When the metasurface is not subject to optical pumping, a plane wave that strikes the metasurface produces a beam perpendicular to the incident wave.

### 2-bit light-controllable digital coding metasurface

**Figure 6:**
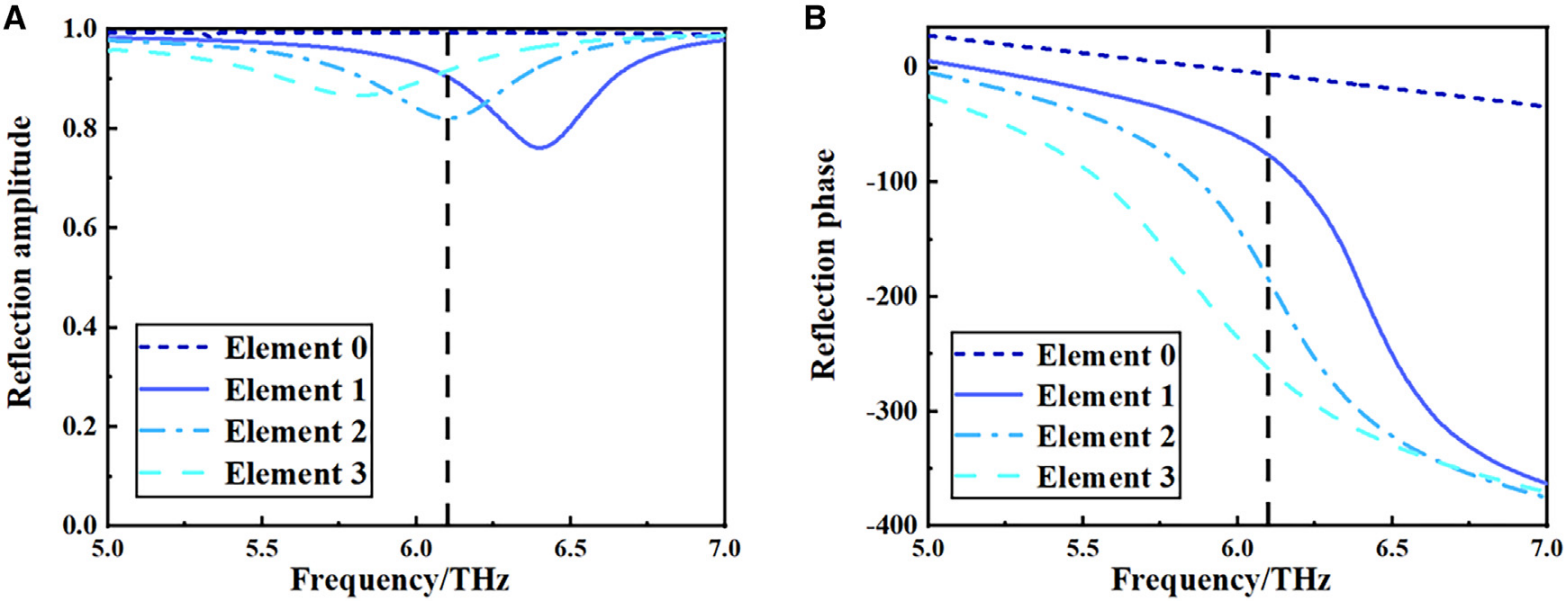
The four digital elements of 2-bit tunable coding metasurfaces and their reflection response curves (A) The reflection amplitude of the 2-bit units from 5 to 7 THz. (B) The reflection phase of the 2-bit units from 5 to 7 THz.

In this paper, we extend the concept of coding metasurfaces from 1-bit coding to 2-bit coding to enhance beam modulation flexibility. 2-bit coding offers a higher degree of freedom to manipulate electromagnetic waves than 1-bit coding. The 2-bit coding units are displayed in Table 3, detailing their structure and phase response. The digital units “0”, “1”, “2”, and “3” have b-sizes of 19.8 μm, 32.7 μm, 35 μm, and 38 μm, respectively, with corresponding digital bits representing phase responses of 0, π/2, π, and 3π/2. The absolute value of the phase is insignificant for coding metasurfaces, it is the difference of the phase that matters. According to Figure 6A, the amplitudes of the “0”, “1”, “2”, and “3” structures at 6.1 THz are 0.99, 0.87, 0.82, and 0.92, respectively. All these amplitudes are above 0.8, indicating that the coded ordering of the metasurface generates a promising electromagnetic response. The phase parameters are shown in Figure 6B, and the phases of the four cells are −5.7°, −94.9°, −185°, and −275°, respectively, and the phase difference between two neighboring cell structures is close to 90°, which is good for 2-bit phase coding near 6.1 THz.

**Table 3:** 2-bit digital elements of tunable coding metasurfaces

Phase response over frequency at 6.1 THz (2-bit)				
Code	“0”	“1”	“2”	“3”
Phase[deg]	−5.7	−94.9	−185	−275.6

**Figure 7:**
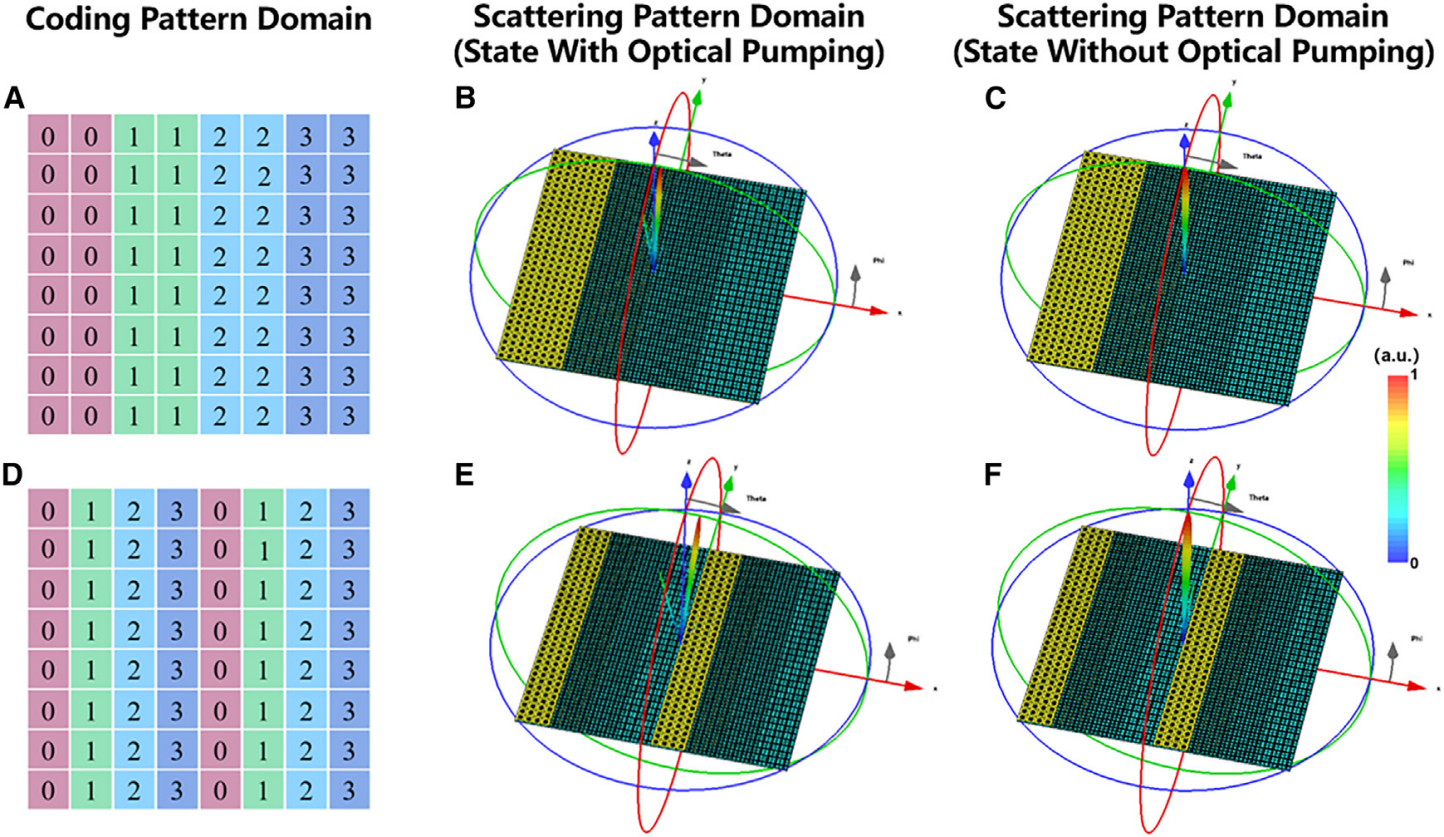
Schematic of and the switching effect of simulated 3D scattering patterns at a 2-bit light-controllable coding metasurface (A) Schematic of the coding sequence 0101 …/0101 … at 6.1 THz. (B) 3D scattering pattern with optical pumping of the coding sequence in Figure 7A. (C) 3D scattering pattern without optical pumping of the coding sequence in Figure 7A. (D) Schematic of the coding sequence 0101 …/1010 … at 6.1 THz. (E) 3D scattering pattern with optical pumping of the coding sequence in Figure 7D. (F) 3D scattering pattern without optical pumping of the coding sequence in Figure 7D.

As demonstrated in Figures 7A and 7B, the deflection angle of the reflected beam from the metasurface in the optically pumped state is approximately 4.4° when the coding sequence is 00-11-22-33. When the coding sequence period is Γ=1280μm, the deflection angle can be calculated using the formula θ=2.19°. The simulation results are consistent with the calculated theoretical results. In the absence of optical pumping, a plane wave hitting the metasurface produces a perpendicular beam opposite to the incident wave. The far-field resultant is plotted as shown in Figure 7C. Similarly, other 2-bit coding metasurface coding sequences 0123/0123 were designed as illustrated in Figure 7D. As illustrated in Figure 7E, the reflected beam exhibits a deflection angle of approximately 2° relative to the positive z axis direction when the metasurface is in the optical pumping state. When the coding sequence period is Γ=640μm, the theoretical calculation results in a deflection angle of θ=2.19°. When the metasurface is without optical pumping, plane wave irradiation of the metasurfaces produces a reflected beam perpendicular to the incident wave, and the far-field resultant is plotted in Figure 7F. The results indicate that the metasurface can be designed to deflect the reflected beam to a specific angle by altering the coding period.

### Convolution operations

Thanks to the Fourier transform the relationship between the coding pattern of the coding metasurface and its far-field direction map, this thesis employs the convolution theorem in signal processing to modulate the far-field direction map. By superimposing another gradient coding sequence onto the existing coding pattern, the far-field radiation direction map can be deflected toward a certain design direction. Rotating the far-field directional map to a larger angle is akin to shifting a baseband signal to a high-frequency carrier in a Fourier transform. The far-field directional map can be deflected to multiple directions with minimal loss by superimposing different periodic coding patterns. This demonstrates the potent and versatile control of electromagnetic waves through the devised coding scheme.

**Figure 8:**
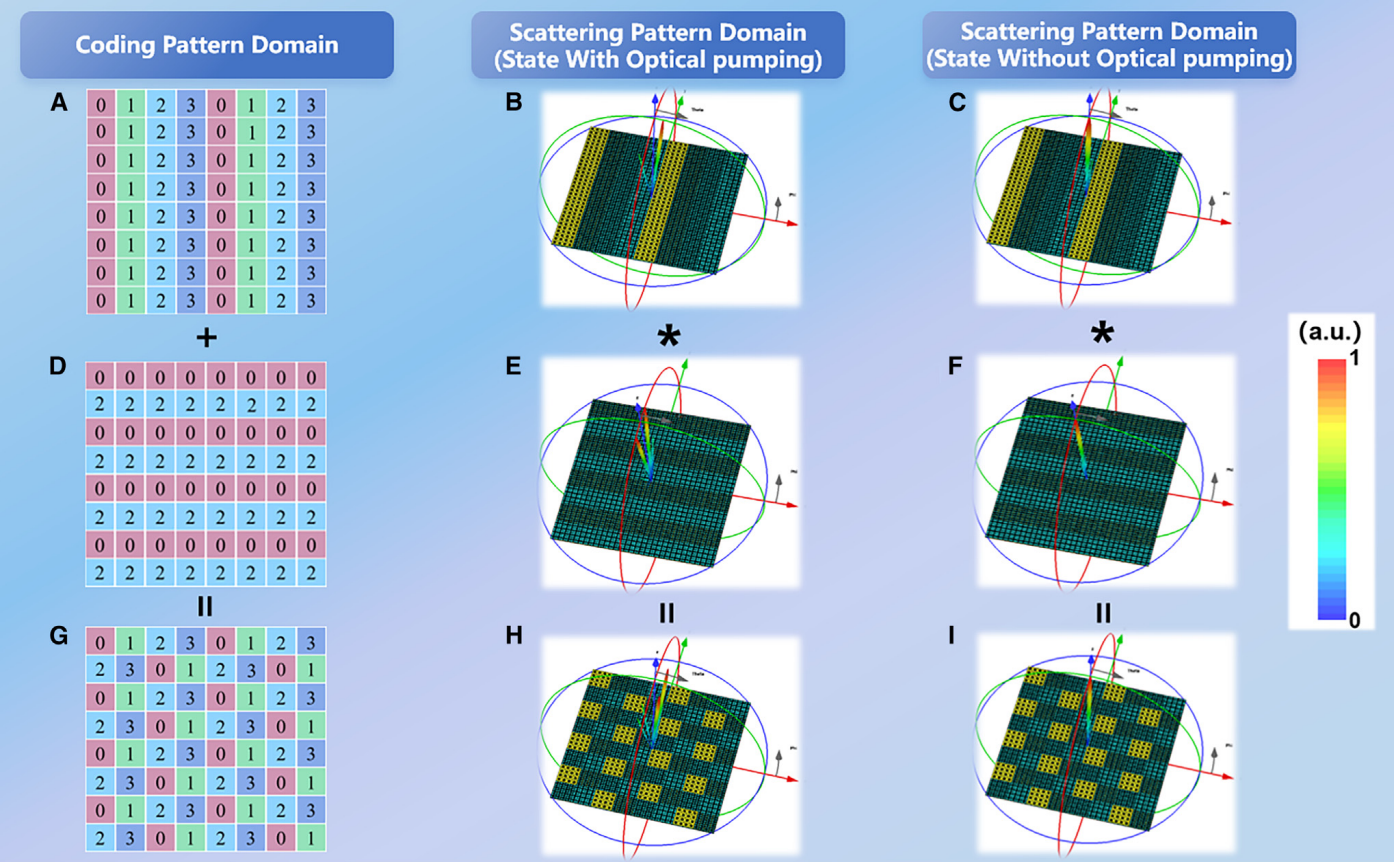
Schematic diagram of the 2-bit tunable coding metasurface convolution operation (A, D, and G) Schematic arrangement of the metasurface units of S1, S2, and S3. (B, E, and H) 3D far-field scattering diagram of S1, S2, and S3 in the optical pumping state. (C, F, and I) 3D far-field scattering diagram of S1, S2, and S3 in the absence of optical pumping.

In this paper, we provide a demonstration of the convolution operation on varied coding sequences utilizing a 2-bit coding metasurface as an illustration. The coding sequence S1 (01230123 …) is a coding metasurface aligned periodically along the x-direction, while the coding sequence S2 (0202 …) is aligned periodically along the y-direction. As shown in Figures 8A, 8D, and 8G, the designed combined coding sequence S3 consists of a superposition of S1 and S2. When the metasurface is irradiated by the pump light, the photosensitive silicon becomes excited. On the incidence of the terahertz wave on the coding metasurfaces S1, the reflected beam is deflected at 6.1 THz and is shown in Figure 8B as a corresponding three-dimensional (3D) scattering pattern. Similarly, in the case of the coding metasurfaces S2, the terahertz wave splits into two symmetric reflected beams at 6.1 THz, and the corresponding three-dimensional (3D) scattering pattern is shown in Figure 8E. As shown in Figure 8F, the far-field scattering pattern of the combined coding sequence S3 consists of the superposition of the far-field scattering patterns of the two coding sequences S1 and S2. As can be seen from the Figure 8, after convolving the double beam coding sequence S2 with the single beam coding sequence S1, the entire double beam can be deflected in the direction of the single beam. Figures 8C and 8F show the far-field scattering diagrams of the S1 and S2 coding sequences when the coding metasurface is irradiated without pump light. As can be seen from Figures 8C and 8F, the far field scattering pattern of both coding sequences is a single vertically reflected beam. As shown in Figure 8I, the two coding sequences are further subjected to a convolution operation, and the convolved far-field scattering also results in a vertically reflected beam.

### OAM generation

In this study, we construct 2-bit coding metasurfaces that are capable of exciting vortex electromagnetic beams within the terahertz frequency band. The generation of vortex beams by coding metasurfaces is achieved through the introduction of an azimuthal phase dependence, designated as $e^{-il\phi }$, into the radiated wave. This modulates the phase distribution into a helical phase profile, which is controlled by the following equation^54^:

(Equation 3) $$\phi_{1}\left(x,y\right)=l\;\tan^{-1}\left(y/x\right)$$

where l is an arbitrary integer according to the requirement of design.

**Figure 9:**
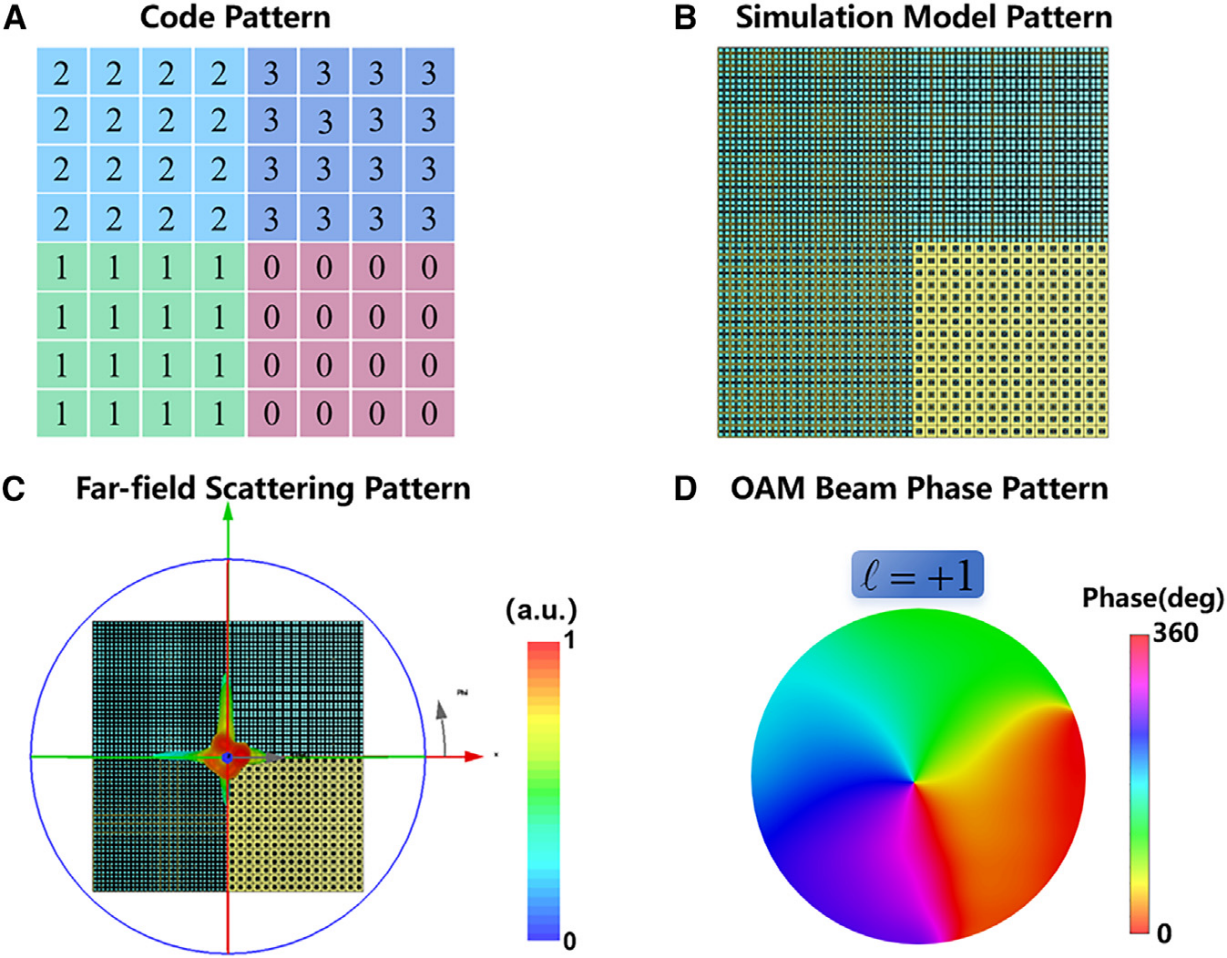
Code patterns and simulated phase distributions for OAM mode l=+1 (A) Quantized code distributions for the OAM mode. (B) Simulation model pattern for the OAM mode. (C) The simulated far-field radiation pattern of the vortex beam generated by coding metasurface at 6.1 THz. (D) Phase distributions OAM mode l=+1.

**Figure 10:**
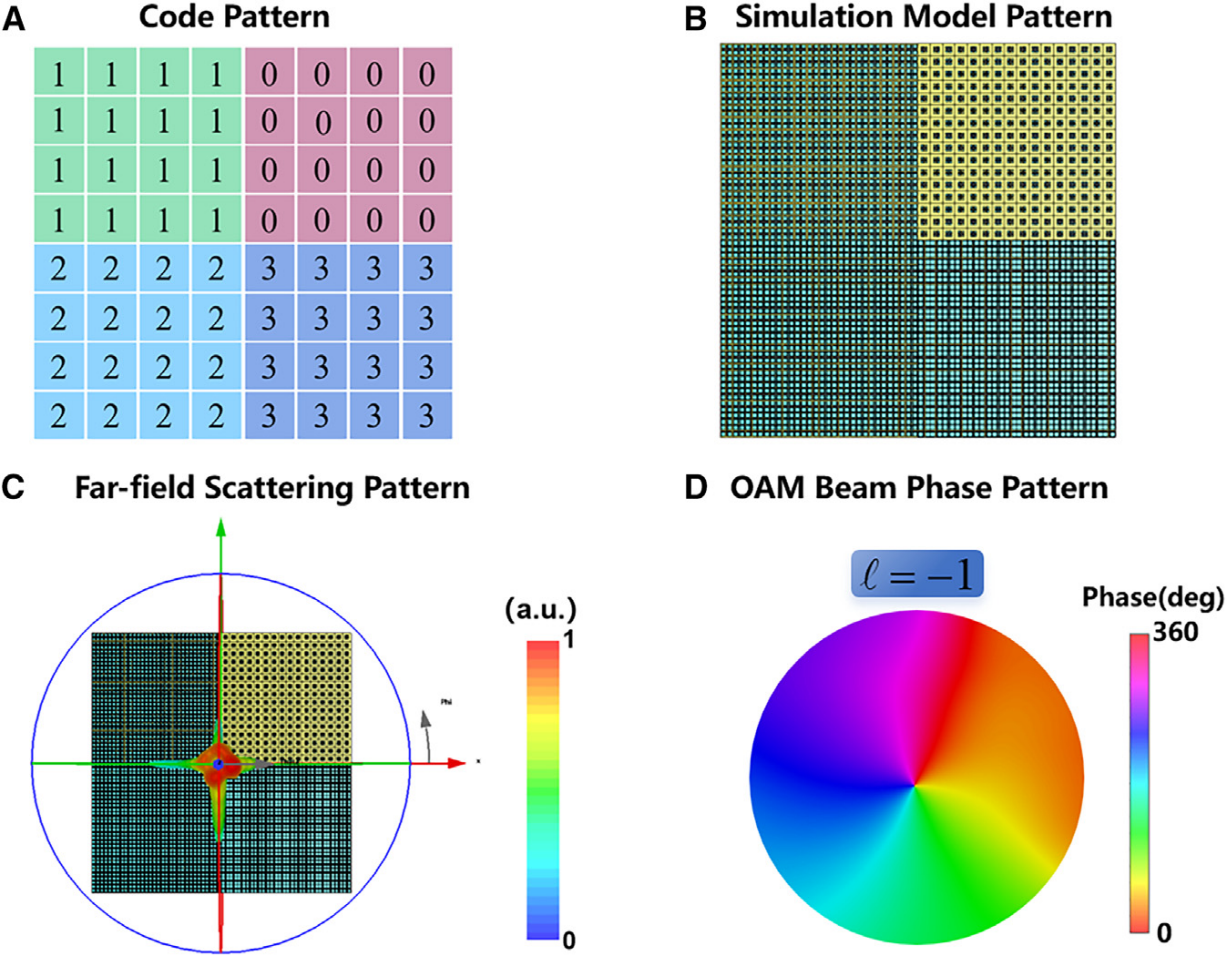
Code patterns and simulated phase distributions for OAM mode l=−1 (A) Quantized code distributions for the OAM mode. (B) Simulation model pattern for the OAM mode. (C) The simulated far-field radiation pattern of the vortex beam generated by coding metasurface at 6.1 THz. (D) Phase distributions OAM mode l=−1.

To generate this helical phase distribution, a 2-bit coding metasurface with four regions with phase shifts ranging from 0 to 2π was constructed, in which 32 × 32 metasurface cells were used, as shown in Figures 9A, 9B, 10A, and 10B). The far-field scattering patterns of the vortex coding metasurface for a perpendicular incident plane wave are illustrated in Figures 9C and 10C. At a frequency of 6.1 THz, the intensity distribution of the vortex beam is circular. Figures 9D and 10D illustrate the simulated phase results for the 6.1 THz orbital angular momentum (OAM) beam, wherein the helical phase pattern is distinctly discernible in both instances. It can be seen that the phase distribution of the two beams conforms to the typical characteristics of OAM beams.

### A genetic algorithm-based approach to terahertz wideband RCS reduction

The primary objective of achieving radar stealth is to reduce the target radar echo signal. The measurement of the strength of the target radar echo is primarily conducted with regard to the radar scattering cross-section (RCS). It is of great significance to recognize the importance of achieving a wide-band RCS reduction in the terahertz band. The fundamental premise of the coding metasurface approach is to disperse the energy of an electromagnetic wave in multiple directions. This, in turn, leads to the conclusion that the energy of each scattered wave is relatively insignificant, with its bandwidth characteristics primarily influenced by the bandwidth of the constant reflection phase difference across different units. The implementation of an inverse design of array coding utilizing genetic algorithms can facilitate a more flexible regulation of electromagnetic waves. Furthermore, the genetic algorithm exhibits robust global search capabilities, which not only enhance the efficiency of the design process but also yield superior outcomes. In this paper, the far-field scattering principle of coding metasurfaces is employed to reverse design the array configuration using genetic algorithms, thereby obtaining the requisite metasurface array code.

**Figure 11:**
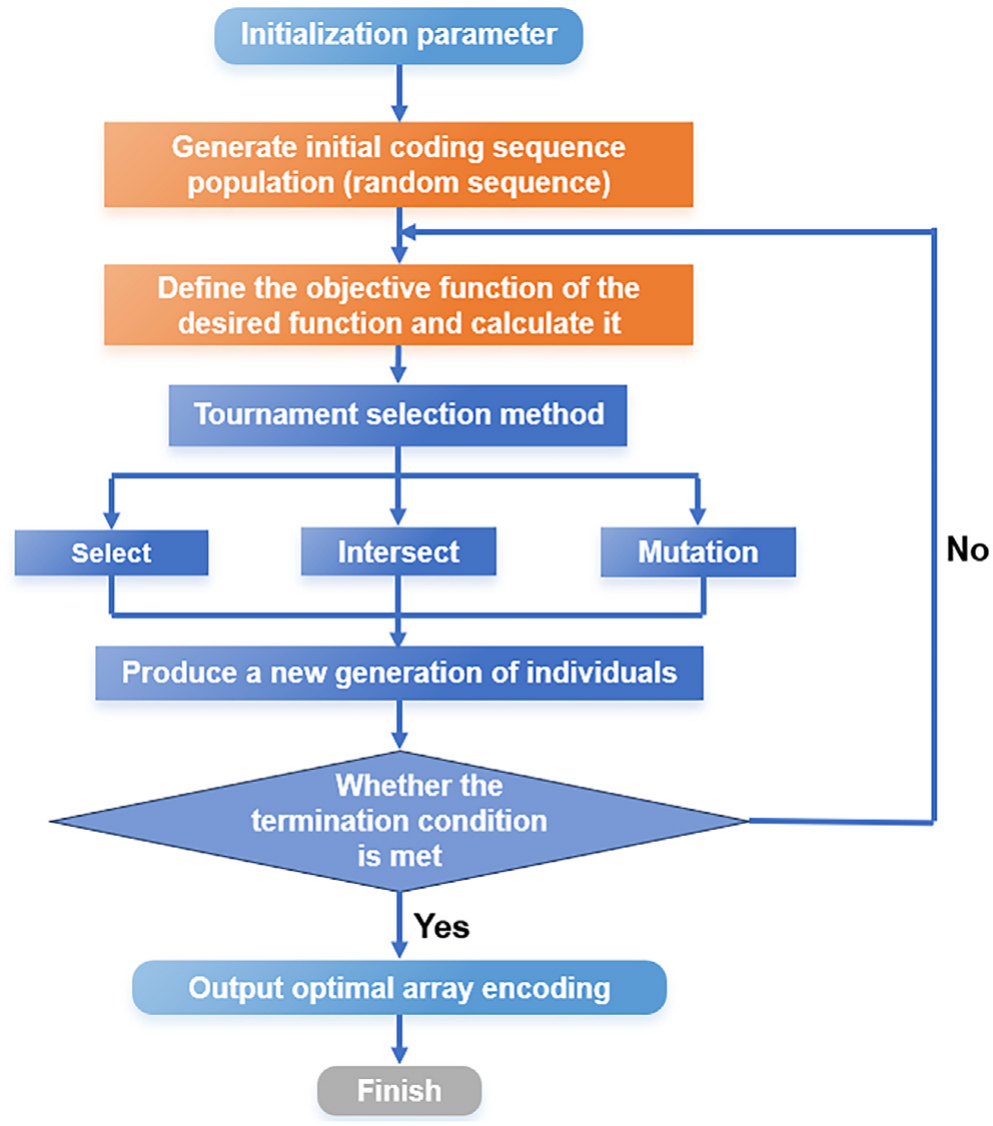
Flow chart of reverse design array coding based on genetic algorithm

The flowchart for the reverse design of metasurface array coding based on a genetic algorithm is presented in Figure 11. This initializes the parameters, including cell period, frequency and metasurface array size. An initial population comprising 50 individuals, each of which is a 32 × 32 random coding sequence, is then generated at random. The objective function for RCS is set to $std\left(\sum_{0}^{360}\sum_{0}^{90}f\left(\theta ,\phi \right)\right)$.

The objective function value under the coding sequence should be calculated, the termination condition should be determined (i.e., whether the maximum number of iterations, set at 1500, has been reached), and the optimal coding sequence should be output if the termination condition is satisfied. Otherwise, the population will be subjected to selection, crossover (random multipoint crossover ratio of 0.9), and mutation (probability of 0.5), and the objective function value of the sub-generation of the population should be calculated. This process is repeated until the requisite number of iterations has been completed, at which point the optimal array code is outputted.

**Figure 12:**
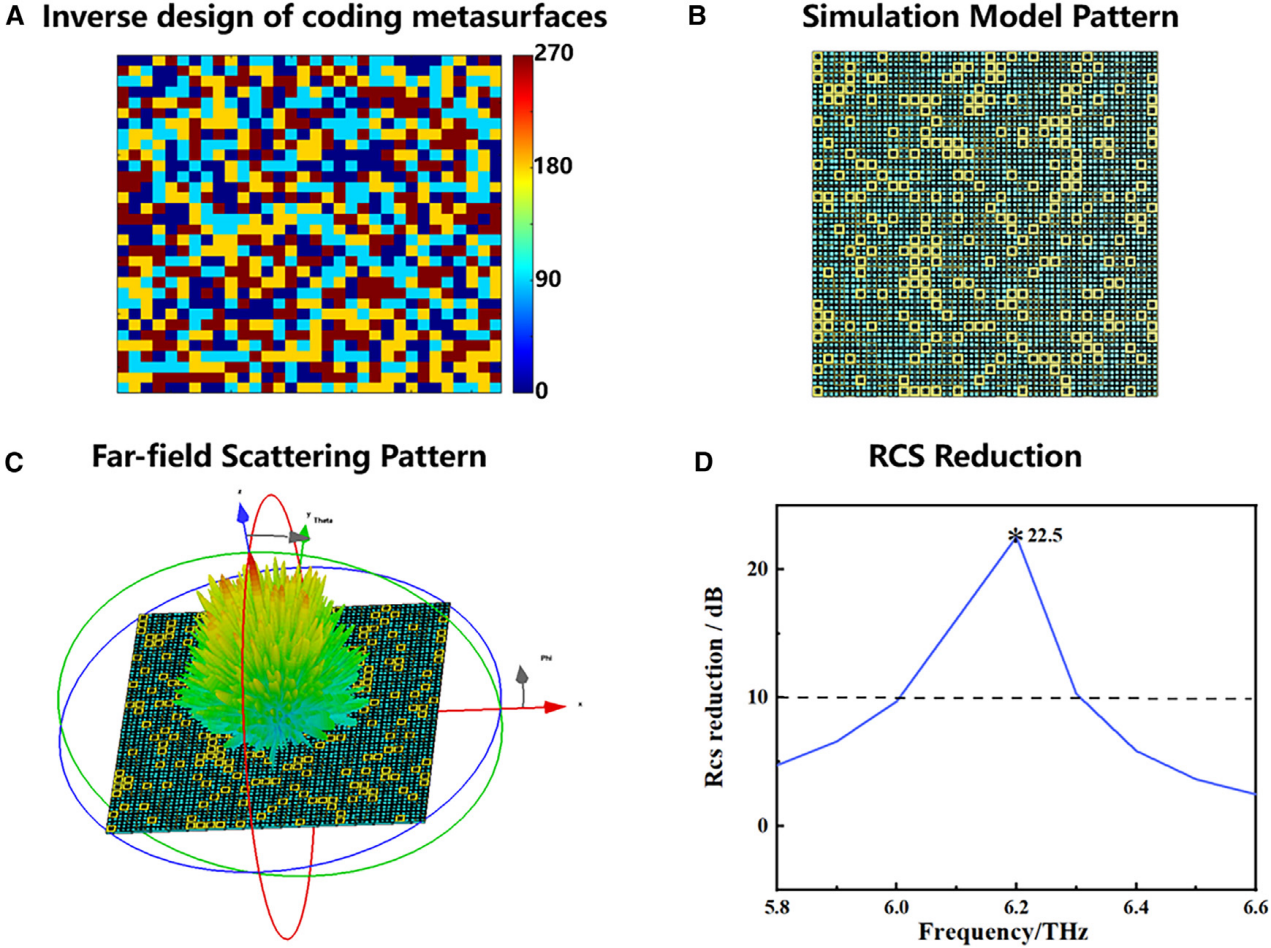
Terahertz wide-band radar scattering cross-section reduction based on wireless light-controllable coding metasurface (A) Coding diagram of the metasurface array obtained by reverse design. (B) Modeling diagram for coding metasurface arrays. (C) 3D far-field scattering maps of coding metasurface. (D) Simulated RCS reduction of designed coding metasurface compared to the metal plate of the same size.

As illustrated in Figures 12A–12C, the coding array generated from the reverse design is initially constructed in business simulation software CST Microwave Studio for simulation purposes, with the objective of obtaining the far-field scattering amplitude. Subsequently, the far-field scattering amplitude of a metal plate of identical dimensions to those of the coding metasurface is calculated in a similar manner. The final step is to compare the RCS values of the two objects in order to ascertain the reduction in RCS. Figure 12D illustrates the numerical curves of the RCS value of the coding metasurface. It can be observed that a considerable 10 dB reduction in RCS can be attained within the frequency range of 6.0–6.3 THz, with the maximum reduction of 22.5 dB occurring at 6.2 THz.

It may be observed that the bandwidth of the RCS reduction is essentially the same as that of the four coding units with a phase difference of approximately 90° and a slight discrepancy in reflection coefficients. This can be attributed to the fact that the reflection coefficients of the metasurface units and the reflection phase difference, in combination, exert an influence on the effect of RCS reduction of the coding metasurface. In the case of 2-bit coding metasurfaces, the RCS is contingent upon two factors: firstly, the difference in reflection coefficients of the metasurface units must be as small as possible; secondly, the reflection phase difference must be as close as possible to 90°. In conclusion, the coding metasurface proposed in this paper represents an effective broadband RCS reduction structure of great significance in the context of terahertz radar stealth and related applications.

### Conclusions

In summary, this paper presents a numerical simulation of a wireless optically controlled terahertz tunable coding metasurface. The metasurface is composed of an upper layer of metal and photosensitive silicon, a dielectric layer, and a metal grounding layer. The structure of the metasurface is simple and there is no need to alter its shape, size, or direction. Light control is used to adjust the conductivity of the photosensitive silicon, achieving a switching effect from a single beam to two or four deflected beams. Convolution operations with different coding sequences can result in varying coding periods, allowing for unrestricted scanning of the terahertz beam in half-space. Furthermore, we have developed a dynamic control design for vortex electromagnetic waves based on wireless optical control coding metasurfaces. Conversely, the array configuration of the coding metasurface has been redesigned using a genetic algorithm, resulting in a reduction of over 10 dB in the radar cross-section within the frequency range of 6.0 THz to 6.3 THz. This work presents a design for terahertz tunable coding metasurfaces and provides a useful reference for terahertz metasurface applications.

### Limitations of the study

The fabrication and performance measurements are the key challenges because of the lack of experimental conditions. According to the previous work, the meta-atom structures can be possibly fabricated as follows: (1) copper is deposited onto the silicon wafer as a bottom plate. (2) A layer of photosensitive silicon is deposited on the SiO_2_ substrate by reactive magnetron sputtering. (3) The pattern of the photosensitive silicon patch is formed by the lithography and reactive ion etching. (4) The layer of copper is deposited on the bottom of the SiO_2_ substrate, and the copper pattern is fabricated on the top surface of SiO_2_ by the second lithography and metallic deposition. The commercial THz system can characterize the optical response of a fabricated device. In future studies, we may seek for the cooperation for the device fabrication and measurement.

## Resource availability

### Lead contact

Further information and requests for resources and reagents should be directed to and will be fulfilled by the lead contact, Min Jia (jiamin@hit.edu.cn).

### Materials availability

This study did not generate new reagents.

### Data and code availability

• Data reported in this paper will be shared by the lead contact upon request.
• This paper does not report original codes.
• Any additional information required to reanalyze the data reported in this paper is available from the lead contact upon request.

## Acknowledgments

This work was supported by 10.13039/501100001809National Natural Science Foundation of China No. 62231012, 10.13039/501100012166National Key Research and Development Program of China under Grant 2021YFB2900500, Natural Science Foundation for Outstanding Young Scholars of Heilongjiang Province under Grant YQ2020F001.

## Author contributions

M.J.: conceptualization, funding acquisition, supervision, resources, writing - review and editing. C.Z.: conceptualization, methodology, simulation and calculation, visualization, validation, writing - original draft, writing - review and editing. H.W.: visualization, writing - review and editing. W.S.: investigation, writing - review and editing. Y.L.: investigation, writing - review and editing.

## Declaration of interests

The authors declare no conflicts of interest.

## STAR★Methods

### Key resources table

**Table:** 

REAGENT or RESOURCE	SOURCE	IDENTIFIER
**Software and algorithms**		

CST	CST China Co.,LTD.	https://www.cst-china.cn
MATLAB	MathWorks Co.,LTD.	https://www.mathworks.com/products/matlab.html

### Experimental model and study participant details

The CST Microwave Studio software has been employed to analyze the far-field patterns of the proposed metasurfaces. In these numerical simulations, the propagation direction of incident wave is set to be perpendicular to the x-y plane where the metasurface.

### Method details

The simulation is conducted with the CST Microwave Studio software. The boundary conditions in the x and y directions are open, and the boundary conditions in the z direction are open (add space). The conductivity of photosensitive silicon before and after transformation is 0S/m and $5.0\times 10^{5}S/m$. The designed coding metasurface consists of an 8×8 array of supersubunits, with each supersubunit comprising a 4×4 grid of metasurface units. Within each supersubunit, all constituent units share the same numerical state, which is indicated by either "0" or "1".

### Quantitation and statistical analysis

The simulation data is produced by CST Microwave Studio software. Figures shown in the main text were produced by Origin and Microsoft PowerPoint from the raw data.

### Additional resources

Any additional information about the simulation and data reported in this paper is available from the lead contact on request.

## References

[1] Tonouchi M. (2007). Cutting-edge terahertz technology. Nat. Photonics.

[2] Song H.J., Nagatsuma T. (2011). Present and future of terahertz communications. IEEE Trans. Terahertz Sci. Technol..

[3] Liu X., Tyler T., Starr T., Starr A.F., Jokerst N.M., Padilla W.J. (2011). Taming the blackbody with infrared metamaterials as selective thermal emitters. Phys. Rev. Lett..

[4] Ko J.H., Yoo Y.J., Lee Y., Jeong H.H., Song Y.M. (2022). A review of tunable photonics: optically active materials and applications from visible to terahertz. iScience.

[5] Xu C., Ren Z., Wei J., Lee C. (2022). Reconfigurable terahertz metamaterials: from fundamental principles to advanced 6G applications. iScience.

[6] Naik K.K. (2024). Asymmetric CPW-fed patch antenna with slits at terahertz applications for 6G wireless communications. Wirel. Netw..

[7] Zhou T., Dong Y., Gong S., Liang S., Ding K., Sun X., Gu S., Zhang B., Yang Z., Zhang Y. (2023). A sub-terahertz high-speed traveling-wave switch modulator based on dynamically tunable double-resonant coupling units. IEEE Trans. Microw. Theory Tech..

[8] Yang S., Ding L., Wang S., Du C., Feng L., Qiu H., Zhang C., Wu J., Fan K., Jin B. (2023). Studying oral tissue via real-time high-resolution terahertz spectroscopic imaging. Phys. Rev. Appl..

[9] Hu W., Xu Z., Han Z., Jiang H., Liu Y., Lu Y., Ligthart L.P. (2022). Ultra-wideband signal generation and fusion algorithm for high-resolution terahertz FMCW radar imaging. Opt Express.

[10] Jia S., Wang S., Liu K., Pang X., Zhang H., Jin X., Zheng S., Chi H., Zhang X., Yu X. (2018). A unified system with integrated generation of high-speed communication and high-resolution sensing signals based on THz photonics. J. Lightwave Technol..

[11] Razavian S., Babakhani A. (2021). Silicon integrated THz comb radiator and receiver for broadband sensing and imaging applications. IEEE Trans. Microw. Theory Tech..

[12] Fang S., Guo W., Huang Y., Shi M., Tian X., Quan B., Xu X., Yi J., Jiang N., Gu C. (2024). Angular dependent terahertz emission from the interplay between nanocrystal diamond film and plasmonic metasurface. iScience.

[13] Yu N., Capasso F. (2014). Flat optics with designer metasurfaces. Nat. Mater..

[14] Zheng X., Lee H., Weisgraber T.H., Shusteff M., Deotte J., Duoss E.B., Kuntz J.D., Biener M.M., Ge Q., Jackson J.A. (2014). Ultralight, ultrastiff mechanical metamaterials. Science.

[15] Yu N., Genevet P., Kats M.A., Aieta F., Tetienne J.P., Capasso F., Gaburro Z. (2011). Light propagation with phase discontinuities: generalized laws of reflection and refraction. Science.

[16] Khorasaninejad M., Capasso F. (2017). Metalenses: versatile multifunctional photonic components. Science.

[17] Zhang X., Qu Z., Wang H. (2020). Engineering acoustic metamaterials for sound absorption: from uniform to gradient structures. iScience.

[18] Jin J., Li X., Pu M., Guo Y., Gao P., Xu M., Zhang Z., Luo X. (2021). Angular-multiplexed multichannel optical vortex arrays generators based on geometric metasurface. iScience.

[19] Zeng Y., Duan Q., Xu J., Yang Z., Chen H., Liu Y. (2023). Tunable circular conversion dichroism of single-layer twisted graphene-patterned metasurface. iScience.

[20] Jiang Z., Liang Q., Li Z., Lv P., Chen T., Li D. (2019). Experimental demonstration of a 3D-printed arched metasurface carpet cloak. Adv. Opt. Mater..

[21] Wang L., Gao F., Teng S., Tan Z., Zhang X., Lou J. (2024). Terahertz tunable vanadium dioxide metasurface for dynamic illusion and cloaking. iScience.

[22] Taher Al-Nuaimi M.K., Whittow W.G., Huang G.L., Chen R.S., Shao Q. (2023). Exploring the EM-wave diffusion capabilities of axicon coding metasurfaces for stealth applications. Opt Express.

[23] Song L.Z., Squires A., Van Der Laan T., Du J. (2024). THz graphene-integrated metasurface for electrically reconfigurable polarization conversion. Nanophotonics.

[24] Tao X., Qi L., Yang J., Uqaili J.A., Lan F., Yang Z. (2023). Bifunctional terahertz metasurface for transmissive broadband linear-to-circular and linear polarization conversion. IEEE Trans. Terahertz Sci. Technol..

[25] Guo W.L., Chen K., Wang G.M., Luo X.Y., Feng Y.J., Qiu C.W. (2020). Transmission--reflection-selective metasurface and its application to RCS reduction of high-gain reflector antenna. IEEE Trans. Antenn. Propag..

[26] Ali A., Khalily M., Brown T., Tafazolli R. (2022). Metasurface-based THz reflectarray antenna with vortex multiplexing and beam-steering capabilities for future wireless communications. iScience.

[27] Yang D., Yuan Y., Wu Q., Zhang K. (2024). High gain OAM antenna with low profile utilizing integrated reflective metasurface. IEEE Antenn. Wireless Propag. Lett..

[28] Guo Y., Zhang Z., Pu M., Huang Y., Li X., Ma X., Xu M., Luo X. (2019). Spoof plasmonic metasurfaces with catenary dispersion for two-dimensional wide-angle focusing and imaging. iScience.

[29] You J.W., Lan Z., Ma Q., Gao Z., Yang Y., Gao F., Xiao M., Cui T.J. (2023). Topological metasurface: From passive toward active and beyond. Photon. Res..

[30] Cui T.J., Qi M.Q., Wan X., Zhao J., Cheng Q. (2014). Coding metamaterials, digital metamaterials and programmable metamaterials. Light Sci. Appl..

[31] Cui T.J., Li L., Liu S., Ma Q., Zhang L., Wan X., Jiang W.X., Cheng Q. (2020). Information metamaterial systems. iScience.

[32] Cui T.J. (2017). Microwave metamaterials—from passive to digital and programmable controls of electromagnetic waves. J. Opt..

[33] Shao R.W., Wu J.W., Wang Z.X., Xu H., Yang H.Q., Cheng Q., Cui T.J. (2024). Macroscopic model and statistical model to characterize electromagnetic information of a digital coding metasurface. Natl. Sci. Rev..

[34] Xiao Q., Ma Q., Ning Y.M., Chen L., Liu S., Zhang J., You J.W., Cui T.J. (2024). Programmable topological metasurface to modulate spatial and surface waves in real time. Nanophotonics.

[35] Li W., Ma Q., Liu C., Zhang Y., Wu X., Wang J., Gao S., Qiu T., Liu T., Xiao Q. (2023). Intelligent metasurface system for automatic tracking of moving targets and wireless communications based on computer vision. Nat. Commun..

[36] Franklin D., Chen Y., Vazquez-Guardado A., Modak S., Boroumand J., Xu D., Wu S.T., Chanda D. (2015). Polarization-independent actively tunable colour generation on imprinted plasmonic surfaces. Nat. Commun..

[37] Zhao H., Wang X., Liu S., Zhang Y. (2023). Highly efficient vectorial field manipulation using a transmitted tri-layer metasurface in the terahertz band. Opto-Electron. Adv..

[38] Gao H., Fan X., Wang Y., Liu Y., Wang X., Xu K., Deng L., Zeng C., Li T., Xia J., Xiong W. (2023). Multi-foci metalens for spectra and polarization ellipticity recognition and reconstruction. Opto-Electron. Science.

[39] Huang Y., Xiao T., Chen S., Xie Z., Zheng J., Zhu J., Su Y., Chen W., Liu K., Tang M. (2023). All-optical controlled-NOT logic gate achieving directional asymmetric transmission based on metasurface doublet. Opto-Electron. Adv..

[40] Li K., Wang J., Cai W., He H., Liu J., Yin Z., Luo D., Mu Q., Gérard D., Liu Y.J. (2022). Electrically switchable structural colors based on liquid-crystal-overlaid aluminum anisotropic nanoaperture arrays. Opt Express.

[41] Fu X., Shi L., Yang J., Fu Y., Liu C., Wu J.W., Yang F., Bao L., Cui T.J. (2022). Flexible terahertz beam manipulations based on liquid-crystal-integrated programmable metasurfaces. ACS Appl. Mater. Interfaces.

[42] Fu Y., Fu X., Yang S., Peng S., Wang P., Liu Y., Yang J., Wu J., Cui T.J. (2023). Two-dimensional terahertz beam manipulations based on liquid-crystal-assisted programmable metasurface. Appl. Phys. Lett..

[43] Cai Z., Liu Y. (2022). Near-infrared reflection modulation through electrical tuning of hybrid graphene metasurfaces. Adv. Opt. Mater..

[44] Efazat S.S., Jam S., Basiri R. (2024). Graphene-based programmable coding metasurface for manipulation of THz wave. Opt Commun..

[45] Huang J., Yin X., Xu M., Liu M., Zhang Y., Zhang H. (2022). Switchable coding metasurface for flexible manipulation of terahertz wave based on Dirac semimetal. Results Phys..

[46] Tian J., Adamo G., Liu H., Wu M., Klein M., Deng J., Ang N.S.S., Paniagua-Domínguez R., Liu H., Kuznetsov A.I., Soci C. (2023). Phase-change perovskite microlaser with tunable polarization vortex. Adv. Mater..

[47] Yang D., Wang W., Lv E., Wang H., Liu B., Hou Y., Chen J.H. (2022). Programmable VO2 metasurface for terahertz wave beam steering. iScience.

[48] Wang H.L., Zhang Y.K., Zhang T.Y., Ma H.F., Cui T.J. (2022). Broadband and programmable amplitude-phase-joint-coding information metasurface. ACS Appl. Mater. Interfaces.

[49] Yang J., Chen J., Quan L., Chen X., Shi H., Liu Y., Xue W. (2022). Flexible beamforming using transmission-type coding metasurface. J. Phys. D Appl. Phys..

[50] Li Z., Wang W., Deng S., Qu J., Li Y., Lv B., Li W., Gao X., Zhu Z., Guan C., Shi J. (2022). Active beam manipulation and convolution operation in VO(2)-integrated coding terahertz metasurfaces. Opt. Lett..

[51] Xiao B., Chen H., Liu J., Yu J., Xiao L. (2023). Multifunctional and tunable terahertz coding metasurfaces based on vanadium dioxide. Opt Commun..

[52] Yang F., Tan T.C., Prakash S., Kumar A., Ariando A., Singh R., Wang N., Pitchappa P. (2024). Reconfigurable wide-angle beam-steering terahertz metasurfaces based on vanadium dioxide. Adv. Opt. Mater..

[53] Jiang H., Sheng L., Luo Y., Meng L., Cao W. (2023). Design of tunable broadband graphene-based metasurface with amplitude-phase modulation. Materials.

[54] Bai G.D., Ma Q., Iqbal S., Bao L., Jing H.B., Zhang L., Wu H.T., Wu R.Y., Zhang H.C., Yang C., Cui T.J. (2018). Multitasking shared aperture enabled with multiband digital coding metasurface. Adv. Opt. Mater..

[55] Ma H., Yang J., Chen T., Duan J., Liu Y., Yang S., Liu L., Gong R., Deng L. (2023). Tunable metasurface for independent controlling radar stealth properties via geometric and propagation phase modulation. Opt Express.

